# microRNA-875-5p plays critical role for mesenchymal condensation in epithelial-mesenchymal interaction during tooth development

**DOI:** 10.1038/s41598-020-61693-w

**Published:** 2020-03-18

**Authors:** Keita Funada, Keigo Yoshizaki, Kanako MIyazaki, Xue Han, Tomomi Yuta, Tian Tian, Kanji Mizuta, Yao Fu, Tsutomu Iwamoto, Aya Yamada, Ichiro Takahashi, Satoshi Fukumoto

**Affiliations:** 1 Section of Orthodontics and Dentofacial Orthopedics, Division of Oral Health, Growth and Development, Kyushu University Faculty of Dental Science, Fukuoka, Japan; 2 Department of Pediatric Dentistry, Tokushima University Graduate School, Tokushima, Japan; 3 Division of Pediatric Dentistry, Department of Oral Health and Development Sciences, Tohoku University Graduate School of Dentistry, Sendai, Japan; 4 Section of Pediatric Dentistry, Division of Oral Health, Growth and Development, Kyushu University Faculty of Dental Science, Fukuoka, Japan

**Keywords:** Morphogenesis, Non-coding RNAs

## Abstract

Epithelial-mesenchymal interaction has critical roles for organ development including teeth, during which epithelial thickening and mesenchymal condensation are initiated by precise regulation of the signaling pathway. In teeth, neural crest-derived mesenchymal cells expressed PDGF receptors migrate and become condensed toward invaginated epithelium. To identify the molecular mechanism of this interaction, we explored the specific transcriptional start sites (TSSs) of tooth organs using cap analysis of gene expression (CAGE). We identified a tooth specific TSS detected in the chromosome 15qD1 region, which codes microRNA-875 (mir875). MiR875-5p is specifically expressed in dental mesenchyme during the early stage of tooth development. Furthermore, PRRX1/2 binds to the mir875 promoter region and enhances the expression of mir875. To assess the role of miR875-5p in dental mesenchyme, we transfected mimic miR875-5p into mouse dental pulp (mDP) cells, which showed that cell migration toward dental epithelial cells was significantly induced by miR875-5p via the PDGF signaling pathway. Those results also demonstrated that miR875-5p induces cell migration by inhibiting PTEN and STAT1, which are regulated by miR875-5p as part of post-transcriptional regulation. Together, our findings indicate that tooth specific miR875-5p has important roles in cell condensation of mesenchymal cells around invaginated dental epithelium and induction of epithelial-mesenchymal interaction.

## Introduction

Tooth morphogenesis, initiated by reciprocal interactions between the ectoderm and neural crest-derived mesenchyme^1–3^, is a good model for understanding the molecular mechanism of epithelial-mesenchymal interaction, because of the well-defined developmental stages and distinctive cell types. In mice, that morphogenesis is initiated by thickening of dental epithelium to form the dental placode, followed by invagination into mesenchyme on embryonic day (E)11.5. In addition, neural crest-derived mesenchymal cells migrate and condense toward the dental epithelial placode. Thereafter, mesenchyme condenses around the epithelial tooth bud and gains an ability for tooth morphogenesis via expression of a particular set of transcription factors and signaling molecules^3,4^. During tooth morphogenesis, invaginated epithelium and condensed mesenchymal tissues interact with each other (epithelial-mesenchymal interaction), and form the shape of the tooth. Finally, dental epithelium and mesenchyme differentiate into ameloblasts, which secretes enamel, and odontoblasts, secreting dentin. However, the molecular mechanism of mesenchymal cell migration and condensation have yet to be clearly elucidated.

Cranial skeletal tissues including tooth, bone, nerves, and blood vessels are mainly derived from neural crest cells (NCCs)^5–7^. The neural crest is composed of transient embryonic tissues present during neural tube formation, whose cells have a high potential for migration and differentiation^7–9^. Disruption of cranial NCC migration during embryonic development can lead to various pathologies, including craniofacial abnormalities, heart malformation, and colonic aganglionosis^10–12^, and numerous studies have implicated the involvement of several families of growth factors, including FGF, BMP, and Wnt, in development of NCCs^13–16^. Furthermore, the PDGF pathway involved in formation of neural crest derivatives is associated with control of NCC migration and proliferation^17–19^, while the PDGF receptor is a marker molecule of cranial NCCs^20–22^.

In the present study, we used cap analysis of gene expression (CAGE)^23^ to identify tooth-specific transcription start sites (TSSs) in order to elucidate the molecular mechanism of tooth development, especially epithelial-mesenchymal interaction. Previously, the functional annotation of the mammalian genome 5 (FANTOM5) project (http://fantom.gsc.riken.jp/5) performed CAGE with 975 human and 399 mouse samples, including primary cells, tissues, and cancer cell lines, which provided comprehensive expression profiles and functional annotation of mammalian cell-type-specific transcriptomes^24^. However, there are no tooth samples in the CAGE libraries developed by the project. CAGE data obtained in the present study were uploaded to ZENBU (http://fantom.gsc.riken.jp/zenbu), based on the genome browser concept, which allowed us to interactively explore the relationship of the genomic distribution of CAGE with expression profiles^25^. Using comprehensive analysis, we identified a tooth specific TSS in the chromosome 15qD1 region that encodes mir875.

In this study, we examined the role of mir875 expression in tooth germ development as well as the effect of mir875 on mesenchymal cell migration. The results indicate that mir875 may be a critical regulator of dental mesenchymal migration and condensation, which is regulated by the PDGF signaling pathway via epithelial-mesenchymal interaction during tooth morphogenesis.

## Results

### MiR875-5p specifically expressed in developing teeth

**Figure 1:**
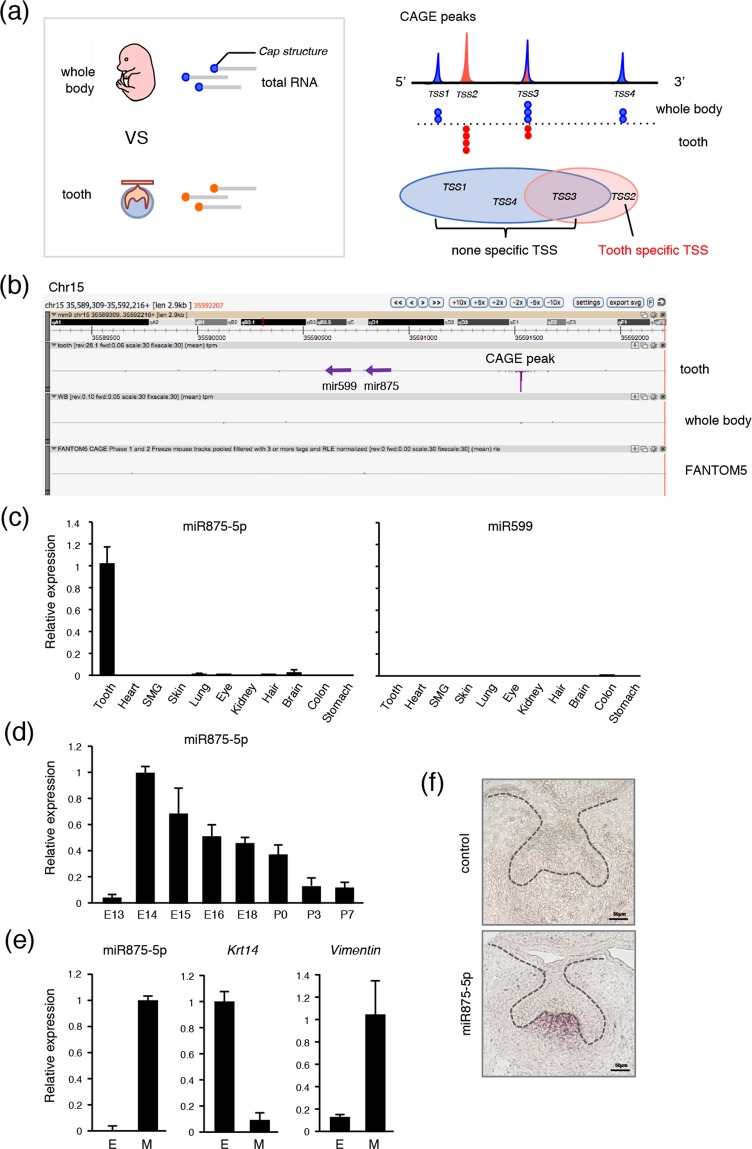
High level of miR875-5p expression in teeth. (**a**) The cap structures of total RNA obtained from E14 tooth and whole body specimens were subjected to CAGE analysis. Tooth-specific TSS was identified as TSS2 in comparison with whole embryos. (**b**) ZENBU results showed that the identified TSS potentially codes mir875 and mir599, which are highly expressed in teeth in comparison with the whole body. The entire FANTOM5 dataset showed no peak in the TSS region. (**c**) qRT-PCR analysis of miR875-5p and miR599 expressions in tooth, heart, submandibular gland, skin, lung, eye, kidney, hair, brain, colon, and stomach specimens from E14 embryos after normalization to *Gapdh* expression. (**d**) qRT-PCR analysis of miR7875-5p expression in teeth obtained from E13 to P7 after normalization to *Gapdh* expression. (**e**) qRT-PCR analysis of miR875-5p, *Krt14*, and *Vim* expressions in tooth epithelium and mesenchyme. (**f**) MiR875-5p localization in E14 first mandibular molar specimens detected by *in situ* hybridization. Error bars represent mean ± S.D. E, epithelium; M, mesenchyme.

CAGE was conducted to reveal TSSs. Total RNA was obtained from E14 mice and TSSs in teeth were compared with those in the whole body (Fig. 1a, left). The mRNA cap structure was trapped by CAGE, then each CAGE peak was mapped onto the genome (Fig. 1a, right; red peak represents tooth-specific TSS). Genes related to tooth-specific TSS are listed in Supplementary Table S1. Using comprehensive analysis, we identified a tooth-specific TSS located in the chromosome 15D1 region (Fig. 1b; red peak). To compare this TSS with findings in the FANTOM5 database^24^, we uploaded tooth CAGE data to the ZENBU genome browser and found that the TSS on chromosome 15 is highly peaked in teeth, whereas no peak is shown in the FANTOM5 CAGE data, revealing this TSS to be highly specific for tooth development (Fig. 1b). We also identified that this TSS potentially codes mir875 and mir599 (Fig. 1b). Together, these results indicate that the identified TSS encodes tooth-specific miRNA, which may play important roles in tooth development.

To confirm whether mir875 and mir599 are transcribed from the identified TSS, qRT-PCR was performed using total RNA obtained from tooth, heart, submandibular gland (SMG), skin, lung, eye, kidney, hair, brain, colon, and stomach samples obtained from E14 embryos. MiR875-5p expression was found to be extremely higher in teeth as compared to the other organs, whereas miR599 showed nearly no expression in any organs from this embryo stage (Fig. 1c). We also confirmed miR875 and miR599 expression in tooth using RNA-sequence (data not shown). Next, we examined the expression of miR875-5p during the stages of tooth development from E13 to E18, as well as on postnatal day (P)0 to P7, which revealed a high level of miR875-5p expression on E14, which gradually decreased thereafter (Fig. 1d). The expression of miR875-5p was relatively low on E13, though remained higher than that in other tissues at this stage. Furthermore, following separation of E14 dental epithelium treated with dispase under a microscope, expression levels were determined by qRT-PCR. The dental epithelium-specific gene *Krt14* was found to be highly expressed in epithelium, while *Vim*, which encodes the mesenchyme-specific protein vimentin, was highly expressed in mesenchyme and miR875-5p was mainly expressed in dental mesenchyme (Fig. 1e). Additionally, *in situ* hybridization was performed using frozen sections of first molars obtained from embryo heads on E14, which revealed localization of miR875-5p in the dental papilla in mesenchyme (Fig. 1f). Together, these results indicate that miR875-5p is specific for developing teeth, and highly expressed in dental mesenchymal cells and localized in the dental papilla in mesenchyme.

### PRRX1/2 enhances mir875 during tooth development

**Figure 2:**
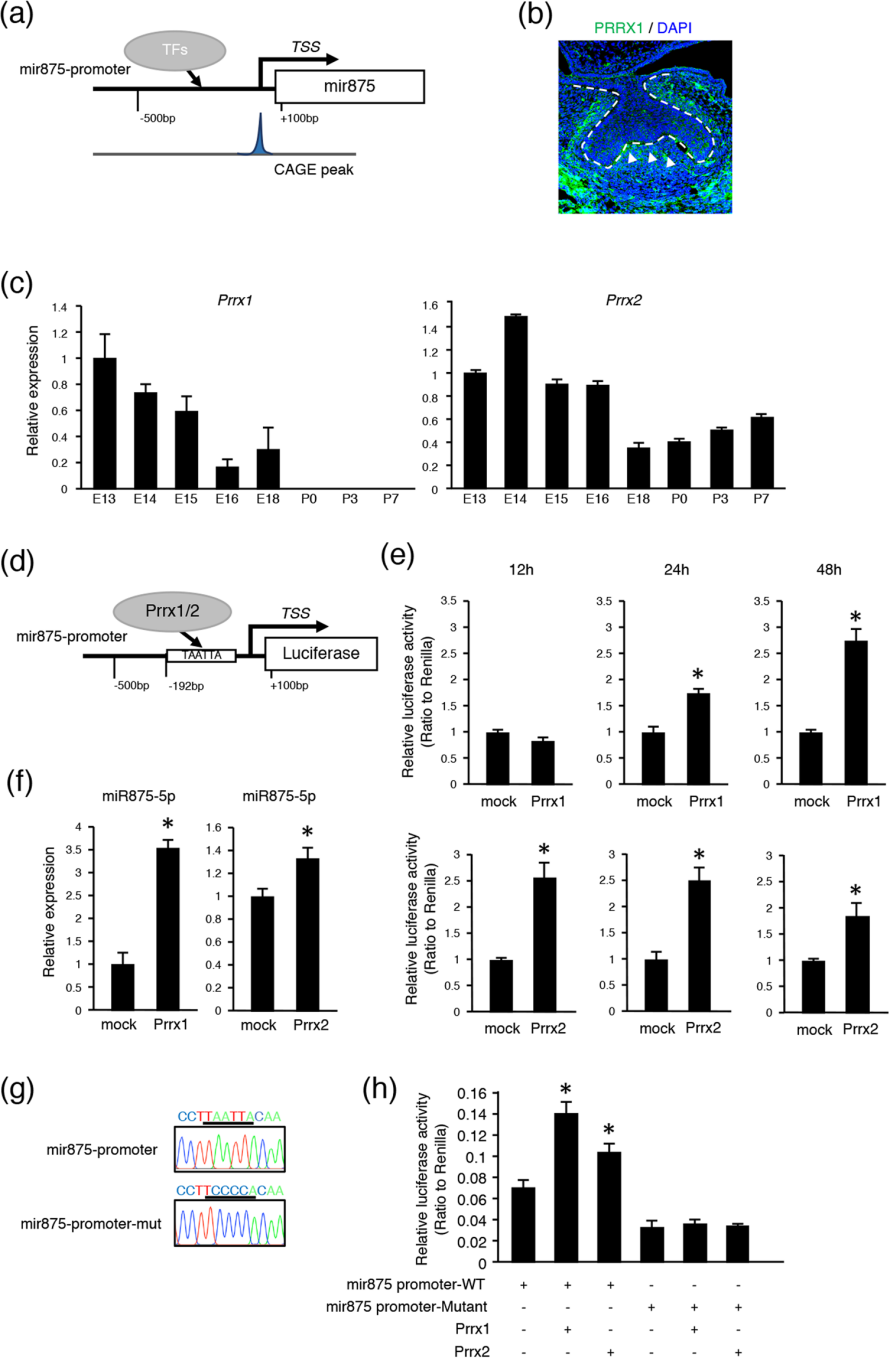
PRRX1 and PRRX2 bind to mir875 promoter region. (**a**) Diagram of mir875 promoter region. The CAGE peak accurately represents TSS. (**b**) Immunohistochemistry image showing PRRX1 (green) and DAPI staining of nuclei (blue) in E14 tooth germ. (**c**) qRT-PCR analysis of *Prrx1* and *Prrx2* expressions in teeth obtained from E13 to P7 after normalization to *Gapdh* expression. (**d**) Schematic diagram showing luciferase reporter vector of mir875 promoter. PRRX1/2 binding was shown to occur in the mir875 promoter region. TAATTA is a potential binding site for PRRX1/2. (**e**) Mir875 promoter reporter activities were evaluated in mDP cells transfected with a *Prrx1/2* expression vector after 12, 24, and 48 hours of culture. (**f**) qRT-PCR analysis of miR875-5p expression in mDP cells cultured with or without *Prrx1/2* transfection. (**g**) Sequences of mir875 promoter and mutant promoter. TAATTA was replaced by the TCCCCA sequence. (**h**) Mir875 promoter reporter activities were evaluated in mDP cells after transfection with a *Prrx1* or *Prrx2* expression vector. *P < 0.05. Error bars represent the mean ± S.D.

The peak of CAGE analysis is able to reveal the TSS of transcribed genes, suggesting that the TSS of mir875 could be accurately predicted. We estimated a region approximately 500 bp upstream to 100 bp downstream from the promoter region of the TSS shown bound by transcription factors (TFs) (Fig. 2a) and also examined potential binding of TFs to the promoter region of mir875 using the JASPAR database. This screening revealed PRRX2 as a candidate molecule, which is known as a homeobox transcription factor (Supplementary Table S2, Fig. 2d). PRRX1 and PRRX2 code two highly similar proteins that share nearly identical homeodomains^26–28^. Our findings confirmed a high level of expression of *Prrx1* during the early stages of tooth development (Fig. 2c), while *Prrx2* was found to be expressed during all developmental stages. Immunohistochemical analysis showed that PRRX1 was highly expressed in dental papilla in mesenchyme (Fig. 2b, arrowhead), which was also shown in a previous report^29^, suggesting expression of both PRRX1 and miR875-5p in the same area (Fig. 1f). Additionally, qRT-PCR findings showed that miR875-5p expression was increased in mouse dental pulpal (mDP) cells with either PRRX1 or PRRX2 overexpression (Fig. 2f). PRRX1/2 can function as a transcription co-activator, and may bind either directly or indirectly to the promoter region of mir875. To further confirm the function of PRRX1/2 in the mir875 promoter, we constructed luciferase reporter vectors after insertion of the mir875 promoter (Fig. 2d). *Prrx1* and *Prrx2* expressing vectors were separately transfected into mDP cells, then promoter activities were evaluated after 12, 24, and 48 hours using a luciferase reporter assay method. *Prrx1* overexpression in dental mesenchymal cells resulted in no difference in transcriptional activity after 12 hours, whereas that was increased after 24 and 48 hours in comparison with the mock group (Fig. 2e). On the other hand, luciferase activity was detectable after 12 hours in PRRX2 transfected cells. Furthermore, we constructed a mutated vector for the PRRX1/2 binding sequence in a reporter assay (Fig. 2g) and transfected that into the cells. The mutant reporter construct showed inhibition of luciferase activity in both PRRX1 and PRRX2 transfected cells (Fig. 2h). Together, these findings indicate that PRRX1/2 function as critical regulators of mir875 and induce transcription of mir875.

### MiR875-5p promotes cell migration in dental mesenchymal cells

**Figure 3:**
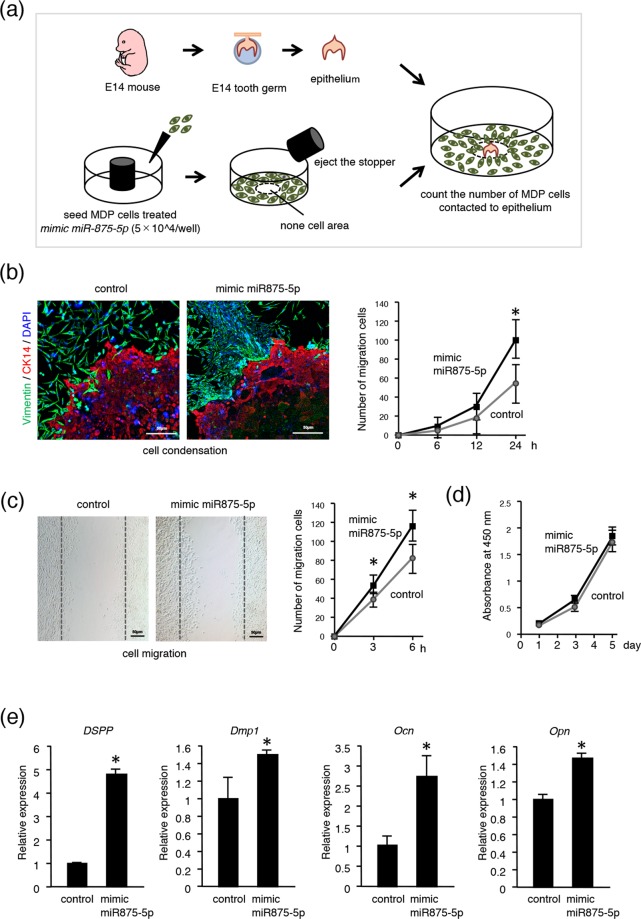
MiR875-5p promotes cell migration in mDP cells. (**a**) General schematic representation of experimental procedure used for cell migration assay. (**b**) The mesenchymal cell marker vimentin (green) and epithelial cell maker CK14 (red) were used for immunohistochemistry. Nuclei were stained with DAPI (blue). Migrated cells were counted after 6, 12, and 24 hours. (**c**) A scratch assay was conducted using mDP cells transfected with the control or mimic miR875-5p. Cell migration numbers were counted after 3 and 6 hours. (**d**) Cell proliferation was analyzed using MTT assay findings after transfection with control or mimic miR875-5p. (**e**) qRT-PCR analysis of expressions of *Dspp*, *Dmp1*, *Ocn* and *Opn* in mDP cells transfected with mimic miR875-5p. *P < 0.05. Error bars represent the mean ± S.D.

Condensation of dental mesenchymal cells to invaginated epithelium is an important phenomenon that occurs during the interaction of epithelial-mesenchymal tissues. To explore the role of miR875-5p in dental mesenchyme, we performed a cell migration assay using mDP cells transfected with mimic miR875-5p or control miRNA. Transfected mDP cells were seeded into 96-well plates with a stopper placed onto each well (Fig. 3a). After 24 hours of culture, the stopper was removed, then dental epithelium samples were dissected from E14 mice embryos and placed into areas without cells (center of well) previously occupied by the rubber stopper (Fig. 3a). After an additional 24 hours of co-culture with tooth germ epithelium samples and mDP cells, immunohistochemistry was performed using the antibody for CK14 (red) and vimentin (green). mDP cells transfected with mimic miR875-5p showed migration toward the CK14-positive dental epithelium to a much greater degree than the control group (Fig. 3b). We counted the number of mDP cells that migrated to dental epithelium after 6, 12, and 24 hours, which showed increases at each time point in the mimic miR875-5p transfected group (Fig. 3b). In addition, a scratch assay of mDP cells was performed after 6 hours of culture, which indicated that the number of miR875-5p expressing cells that migrated to the scratched areas was increased in comparison with the control cells (Fig. 3c). We also evaluated cell proliferation using mDP cells with an MTT assay, but found no significant difference between the mimic miR875-5p treated and control groups (Fig. 3d), indicating that miR875-5p promotes the migration ability of mDP cells and has no effect on cell proliferation. In addition, the effect of miR875-5p on odontoblast differentiation was examined, which showed expression of the dentin markers *Dspp*, *Dmp1*, *Ocn* and *Opn* in miR875-5p transfected cells (Fig. 3e), suggesting an important role for miR875-5p in odontoblast differentiation.

### MiR875-5p promotes cell migration by regulating PDGF signaling pathway

**Figure 4:**
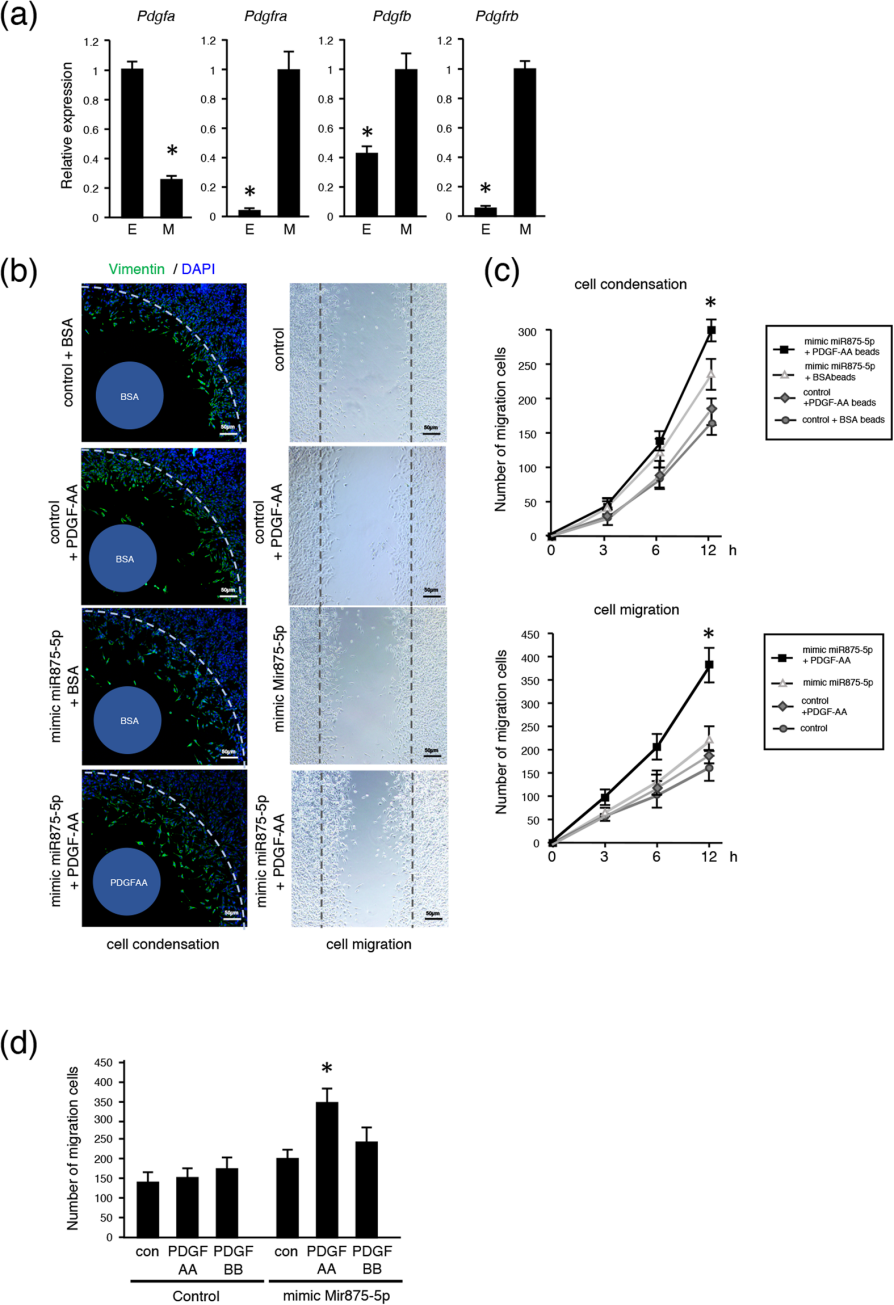
mi875-5p promotes cell migration by activating PDGF signaling pathway. (**a**) qRT-PCR analysis of *Pdgfa*, *Pdgfra*, *Pdgfb* and *Pdgfrb* expressions in E14 tooth epithelium and mesenchyme. (**b**) Migration and scratch assays. The mesenchymal cell marker vimentin (green) was used for immunohistochemistry and nuclei were stained with DAPI (blue). Collagen beads coated with BSA or PDGF-AA were placed in the center of the wells. A scratch assay was conducted using mDP cells transfected with the control or mimic miR875-5p with PDGF-AA optimally added. (**c**) Migrated cell numbers were counted after 3, 6, and 12 hours. (**d**) The numbers of mDP cells transfected with the control or mimic miR875-5p with optimally added PDGF-AA or PDGF-BB were counted after 12 hours. *P < 0.05. Error bars represent mean ± S.D. E, epithelium; M, mesenchyme.

PDGF signaling is essential during embryonic development, and it is especially important to note that its ligand PDGFA is related to NCC migration and the EMT process^30,31^. *Pdgfra*, a PDGF receptor, is also known as a neural crest marker, indicating that PDGF signaling may have a role in dental mesenchymal cell migration. To confirm our speculation, we examined the expression of PDGF signaling molecules in the tooth germ. That of *pdgfa*, which also encodes PDGFA, was higher in tooth epithelium tissues from E14 mice as compared to epithelium from other organs, while *pdgfb*, another gene that encodes PDGFB, showed higher expression in mesenchyme. Furthermore, both PDGFRα and PDGFRβ were found to be expressed in dental mesenchyme at the mRNA level (Fig. 4a). Next, we conducted cell migration (Fig. 4b, left) and scratch (Fig. 4b, right) assays using mDP cells transfected with control miRNA or mimic miR875-5p, with or without PDGF-AA recombinant protein (Fig. 4b). Overexpression of miR875-5p with PDGF-AA enhanced cell migration into PDGF-AA-soaked bead and scratch areas at higher rates as compared to the PDGF-AA-free groups (Fig. 4b,c). When mDP cells transfected with control or mimic miR875-5p were then stimulated with PDGF-AA or PDGF-BB, those transfected with miR875-5p and stimulated with PDGF-AA showed a multiplicative effect for promoting cell migration (Fig. 4d). These results suggest that miR875-5p enhances cell migration by regulating the PDGF signaling pathway, especially PDGF-AA, but not PDGF-BB.

### MiR875-5p promotes cell migration by suppressing PTEN expression

**Figure 5:**
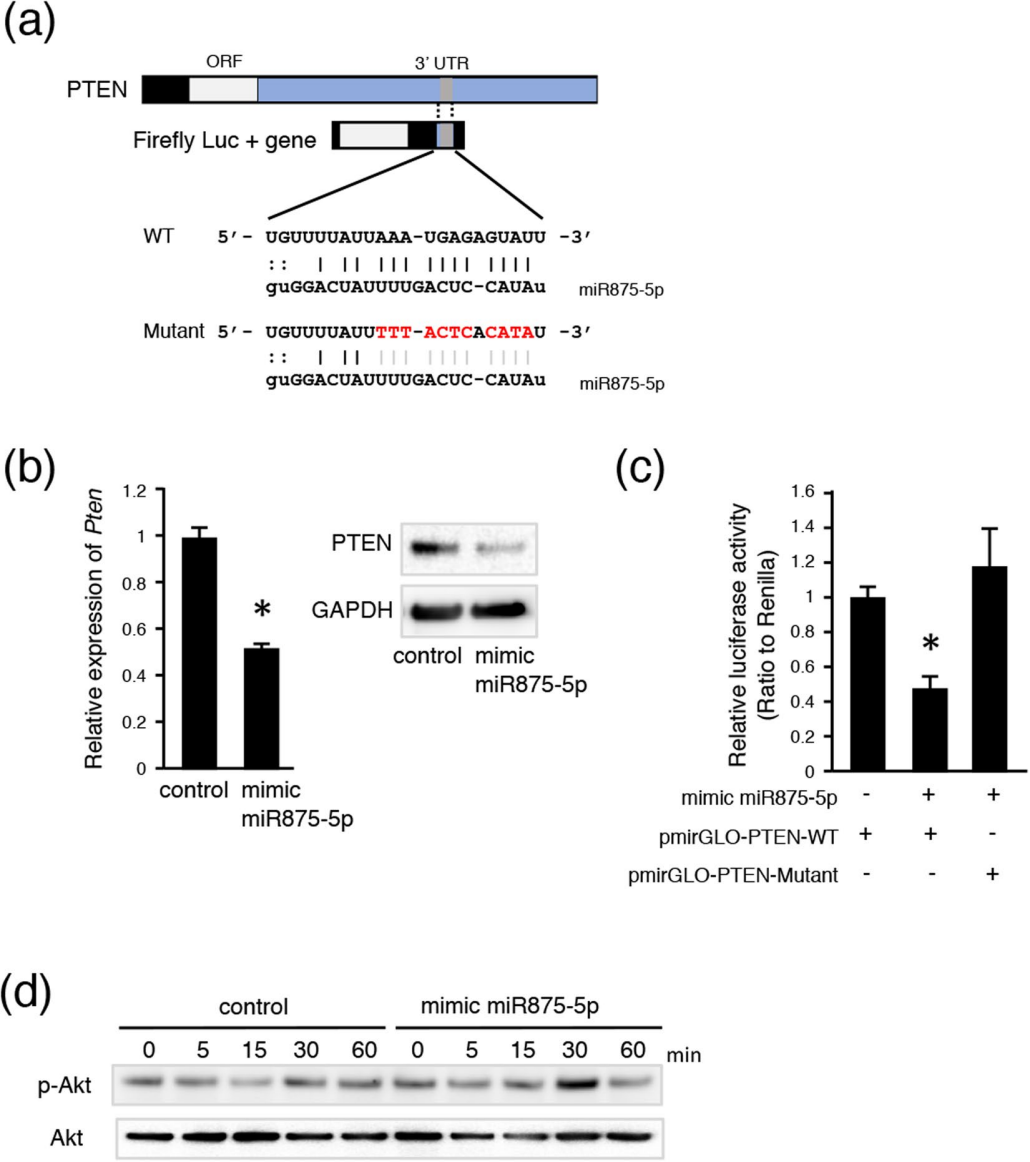
MiR875-5p inhibits *Pten* and regulates phosphorylation of Akt. (**a**) Schematic of reporter construct used to evaluate the role of complementarity between miR875-5p and *Pten* 3′ UTR (blue). The wild-type (WT) construct had a 22-nt fragment of the *Pten* UTR, which included miR875-5p target sites (gray) inserted within the firefly luciferase 3′ UTR. The mutant construct was identical to the WT construct, except that it had several disruptions of pairing to miR875-5p. (**b**) qRT-PCR analysis of *Pten* in mDP cells cultured with the control or mimic miR875-5p after transfection and normalization to *Gapdh* expression. Western blotting results of PTEN and GAPDH in mDP cells cultured with the control or transfected with mimic miR875-5p. The ful-length blots are presented in Supplementaly Fig. 1a. (**c**) *Pten* WT and mutation reporter activities were evaluated in mDP cells transfected with mimic miR875-5p. (**d**) Western blotting of p-Akt and Akt in mDP cells with or without mimic miR875-5p after culturing for 1 hour. The ful-length blots are presented in Supplementaly Fig. 1b.*P < 0.05. Error bars represent the mean ± S.D.

To identify the target gene of miR875-5p in tooth development, we examined the miRNA database (microRNA.org) and predicted *Pten* as a potential target gene, because of a good conserved sequence to bind miR875-5p in humans and mice in the 3′ UTR region (Fig. 5a). qRT-PCR results revealed that the relative expression of *Pten* mRNA was decreased in mimic miR875-5p transfected mDP cells (Fig. 5b), while western blotting analysis showed that miR875-5p inhibited the translation of PTEN in mDP cells (Fig. 5b, Supplementary Fig. S1a). The expression of Pten was found to gradually increase in the process of tooth development (Supplementary Fig. S2a), suggesting that miR875-5p inhibits the expression of Pten in an early developmental stage. To confirm whether the miR875-5p binding motif in PTEN is critical, a 22-nt segment including miRNA target sites was inserted downstream of a firefly luciferase ORF, then luciferase activity was compared to that of an analogous reporter with point substitutions disrupting the target sites (Fig. 5a). Luciferase activity was found to be inhibited by mimic miR875-5p in the *Pten* wild-type sequence, whereas miR875-5p had no effect towards the *Pten* mutant sequence (Fig. 5c). Furthermore, analysis of phosphorylation of Akt, a cross-talk molecule of PTEN, after stimulation of PDGF-AA was conducted, showed that transfection of mimic miR875-5p enhanced phosphorylation of Akt (Fig. 5d, Supplementary Fig. S1b), indicating that a decrease in PTEN protein may accelerate the PI3K-Akt pathway.

### MiR875-5p promotes cell migration by suppressing STAT1 expression

**Figure 6:**
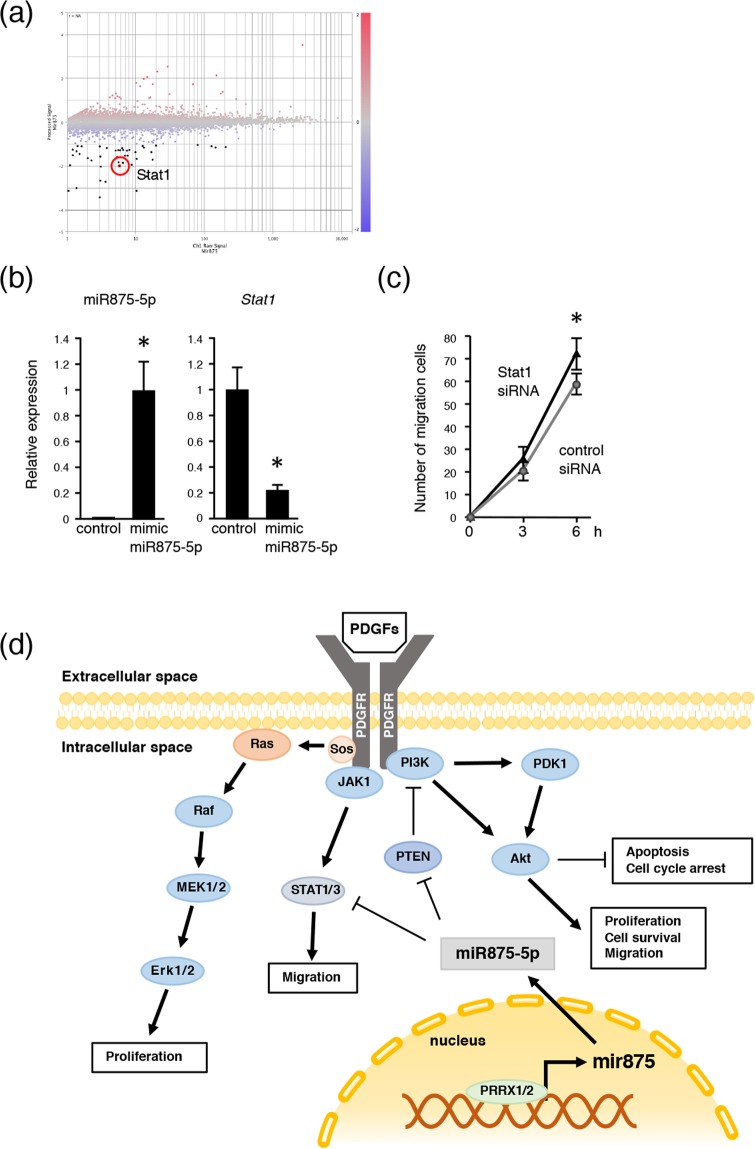
MiR875-5p promotes cell migration by suppressing *Stat1* expression. (**a**) Mimic miR875-5p transfected mDP cells showed differentially expressed genes in CAGE analysis and the results were compared to non-transfected mDP cells. Highlighted plot indicates *Stat1*. Red and blue plots represent up- and down-regulated genes, respectively. (**b**) qRT-PCR analysis of miR875-5p, *Stat1* in mDP cells cultured with the control or transfected mimic miR875-5p after normalization to *Gapdh* expression. (**c**) Scratch assays were conducted using mDP cells transfected with control siRNA or *Stat1* siRNA. The numbers of migrated cells were counted every 3 hours. *P < 0.05. Error bars represent the mean ± S.D. (**d**) Schematic diagram showing roles of miR875-5p in dental mesenchymal cells.

Dental mesenchymal cell condensation was significantly increased in cells expressing miR875-5p, suggesting that cell migration was increased in mimic miR875-5p overexpressing cells. To identify target genes for cell migration, CAGE analysis of miR875-5p overexpressing and control cells was performed, and gene expressions in mimic miR875-5p transfected mDP cells were compared with those of control mDP cells. Up- and down-regulated genes in miR875-5p over-expressing cells are listed in Supplementary Table 3. Scatter plot data revealed that a large number of genes in mDP cells were either up- or down-regulated (Fig. 6a), including *Stat1*, which has been reported to be related to cell migration^32,33^. qRT-PCR results also showed that the expression of *Stat1* mRNA expression was decreased in mimic miR875-5p transfected mDP cells (Fig. 6b). The expression of Stat1 was higher in tooth developmental stages (P0-P7) (Supplementary Fig. S2b), indicating that miR875-5p inhibits the expression of Stat1 during early development. In addition, cell migration following stimulation of *Stat1* siRNA in mDP cells was increased, which was consistent with results noted with miR875-5p stimulation (Figs. 3c and 6c). Thus, the effect of miR875-5p to inhibit STAT1 expression may be important for cell migration increment.

## Discussion

Genome-wide expression analysis is a key approach for rapid and systematic analysis of biological systems. Furthermore, CAGE allows high-throughput identification of sequence tags corresponding to mRNA 5′ ends at cap sites and can identify transcriptional starting points^34,35^. A cDNA microarray was also used for tissue expression analysis, though we were unable to identify the transcriptional starting point or cis regulatory element. This screening method is generally designed to detect exons of known genes and may not include novel transcripts such as noncoding RNAs (ncRNAs). RNA sequencing is a powerful tool for identifying mRNA expression and can reveal comprehensive splicing variants, though it is difficult to identify the transcriptional start point because of the lower level of coverage of the 5′ ends of examined transcripts. Using CAGE analysis, we identified a tooth-specific TSS in the chromosome 15qD1 region that potentially coded mir875 and mir599. Our findings also identified the promoter sequence of mir875. We then examined transcription factors with potential to bind to the promoter region of mir875 using the JASPAR database. In that screening, PRRXs, known as a homeobox transcription factor, was revealed as a candidate molecule.

Reciprocal signaling between dental epithelium and mesenchyme is required for tooth development. The paired related homeobox genes PRRX1 (Prx1) and PRRX2 (Prx2) are expressed in a mesenchymal manner in the tooth germ, and regulate epithelial-mesenchymal interaction^29,36^. A deficiency of *Prrx1* and *Prrx2* results in molar malformations, including cusp changes and ectopic epithelial projections^29^, indicating their important role in tooth development. Our results showed that PRRX1 and PRRX2 bind to the promoter region of mir875, and induce expression of mir875 (Fig. 2). Thus, PRRXs regulate mir875 expression and possibly dental mesenchymal cell differentiation.

Previous studies have found miR875-5p only in cancer cells, and showed that it promotes cancer cell invasion and epithelial-to-mesenchymal transition^37–39^. The present findings revealed expression of miR875-5p in dental mesenchyme, which might be related to the PDGF signaling pathway. In tooth germ samples, PDGF-A and PDGF-B were reported to be expressed in both dental epithelium and dental mesenchyme^40^, while in salivary glands, which are also regulated by epithelial-mesenchymal interaction, PDGF-A has been found to be expressed in epithelium but not mesenchyme^41^. Furthermore, in a previous study, PDGF receptor α and β (PDGFRα and PDGFRβ, respectively) were found expressed in dental and salivary mesenchyme, but not epithelium, indicating cross-talk between epithelium and mesenchyme via PDGF, while PDGF-BB promoted dental mesenchymal cell proliferation and PDGF-AA did not, whereas PDGF-AA induced ameloblastin expression in dental epithelium and ameloblast differentiation^40^. However, the molecular mechanism of the effects of PDGF-AA on dental mesenchyme are not clearly understood. Mice with a *Pdgfa* deficiency have embryonic mortality. Using tooth germ transplantation into a kidney capsule, loss of *Pdgfa* was found to not have effects on proper odontoblast proliferation or differentiation in cranial neural crest-derived mesenchyme, while odontoblast cell organization was disturbed in the cusp forming area, resulting in dental cusp growth defects^42^. The cellular organization of dental mesenchyme is important for tooth morphogenesis, especially tooth cusp formation. PDGFRα signaling was demonstrated to regulate fibroblast migration via Akt and the MEK1/2-ERK1/2 signaling pathway, and PDGF-AA induced fibroblast migration while inhibition of Akt signaling reduced PDGF-AA-induced cell migration^43^. In the present study, PDGF-AA administration alone did not have an effect on cell migration, though transfection of mimic miR875-5p enhanced PDGF-AA-dependent dental mesenchymal migration, indicating regulation of PDGF signaling by miR875-5p. Nevertheless, additional experiments are needed to identify direct regulation of the PDGF signaling pathway by miR875-5p.

Cellular migration is important for organ development in association with cranial NCCs and cancer cell metastasis. PTEN and wnt/b-catenin signaling abnormalities have been strongly implicated in various types of malignant cancer, and shown to regulate cell invasion and migration^44^. The tumor suppressor PTEN gene was first identified in brain, breast, and prostate cancer tissues^45^, then later shown to be a protein/lipid phosphatase that directly antagonizes the activity of phosphatidylinositol-3-kinase (PI3K) to negatively regulate the Akt pathway, which in turn plays a vital role in cell migration^46,47^. The present findings indicate *Pten*, which has a conserved sequence, to be a potential target gene for binding miR875-5p in humans and mice in the 3′ UTR region (Fig. 5a). Interestingly, mimic miR875-5p inhibited the expression of PTEN protein and accelerated PDGF-AA-induced phosphorylation of Akt, indicating that down-regulation of PTEN by miR875-5p has effects on PDGF-AA-induced Akt phosphorylation and cell migration in neural crest-derived dental mesenchymal cells (Fig. 6d).

CAGE analysis also identified the molecular regulation by miR875-5p, as overexpression of mimic miR875-5p resulted in decreased expression of *Stat1*. STATs are regulated by PDGF signaling via Rac1 GTPase, and have effects on cell proliferation and migration^48^, while PDGF activates multiple signaling pathways including PI3-kinase and the MAPK pathway, and the STAT pathway has been shown to interact with both the MAPK and PI3-kinase pathways^49,50^. In the present study, inhibition of *Stat1* resulted in enhancement of cell migration that was similar to overexpression of miR875-5p, thus STATs may also regulate PDGF-induced cell migration and condensation of dental mesenchyme (Fig. 6d).

In summary, the present findings revealed that epithelial PDGF-AA signaling was regulated by mir875 expressing in neural crest-derived dental mesenchyme. Mesenchymal cells expressing miR875-5p showed migration to dental epithelium and then participated in epithelial-mesenchymal interaction. Our results suggest that tooth-specific miRNA may regulate mesenchymal cell condensation to dental epithelium and be important for tooth morphogenesis.

## Methods

### CAGE analysis

Total RNA was isolated from early stage tissues (E14 tooth, whole body) using TRIZOL reagent (Life Technologies) and purified with an RNeasy Mini kit (Qiagen), according to the manufacturer’s protocol. RNA quality was verified using a Bioanalyzer (Agilent), with the results showing an RNA integrity number (RIN) greater than 8.5 in all samples. CAGE analysis was performed by DNAFORM (Yokohama, Japan). Briefly, CAGE tag sequencings in each organ were aligned on the reference genome (mm9), then the mapped reads were converted into a CAGE defined transcriptional start site using SAMtools of the MOIRAI pipeline^51^. Gene expression analysis was performed using Subio Platform, version 1.18 (Subio, Amami, Japan).

### RNA isolation and RT-qPCR analysis

Total RNA was isolated from E14 mouse tissues (tooth, skin, lung, liver, kidney, heart, eye, brain) and molar tooth buds obtained during developmental stages (E11, E13, E14, E15, E18, P0, P3, P7) using TRIZOL reagent (Life Technologies). cDNA was synthesized using SuperScript III reverse transcriptase reagent (Life Technologies) for mRNA. The specific forward and reverse primers used with qRT-PCR for cDNA were as follows: *Krt14*, 5′ -gtacgagaagatggcggaga- 3′ and 5′ -ctttcatgctgagctgggac- 3′; *Vim*, 5′ -cagcagtatgaaagcgtgg- 3′ and 5′ -ggaagaaaaggttggcagag- 3′; *mPdgfra* 5′ -ggaacctcagagagaatcgg- 3′ and 5′ -cagctgaggaccagaaagac- 3′; *mPdgfrb* 5′ -tgtgcagttgccttacgact- 3′ and 5′ -cgctacttctggctgtcgat- 3′; *mPdgfa* 5′ -ttcgtcgataacacgcacga- 3′ and 5′ -ttcccagagtcccctcatgt- 3′; and *mPdgfb*, 5′ -gctgcaggcttctcttgact- 3′ and 5′ -gatctgggtgccatcagagt- 3′; *mStat1*, 5′ -ctcagaaatccgcctgtctc- 3′ and 5′ -acacacgtgccacaaaacat- 3′; *mPten*, 5′ -gaaagggacggactggtgta- 3′ and 5′ -tacatagcgcctctgactgg- 3′; *mPrrx1*, 5′ -cagcaggacaatgaccagt- 3′ and 5′ -gaaaccacacctgcactctg- 3′; *mPrrx2*, 5′ -cgtggcaccaaacgaaagaa- 3′ and 5′ -tggaaccagacttggacacg- 3′; and glyceraldehyde 3-phosphate dehydrogenase (*Gapdh*), 5′ -ggagcgagaccccactaacatc- 3′ and 5′ -ctcgtggttcacacccatcac- 3′; *mDSPP*, 5′ -catgaaacgacgcctcagag- 3′ and 5′ -catcctcctctaccccgttc- 3′; *mDmp1*, 5′ -tcgatcgctcctg- 3′ and 5′ -cagtgaggatgaggcagaca- 3′; *mOcn*, 5′ -gcgctctgtctctctgacct- 3′ and 5′ -gccggagtctgttcactacc- 3′; and *mOpn*, 5′ -ccatctcagaagcagaatc- 3′ and 5′ -atccgagtccacagaatc- 3′. The expression of each gene was normalized to that of *Gapdh*. For miRNA, cDNA was synthesized using an miScript II RT Kit (Qiagen). qRT-PCR was performed using an miScript SYBR Green PCR Kit (Qiagen) with a CFX Connect Real-Time PCR detection system (Bio-Rad). The specific forward primers used for miRNA were obtained using an Mm-mir-875-5p_1 miScript Primer Assay (MS00012747, Qiagen). Expression of miR875-5p was normalized to that of RNU6-2_11 shown in miScript Primer Assay (MS00033740, Qiagen) findings.

### Tissue preparation and *in situ* hybridization

All animal experiments were approved by the ethics committee of Kyushu University Animal Experiment Center (protocol no. A30-100-0) and all procedures were performed in accordance with the relevant guidelines and regulations. E14 mice were euthanized by anesthesia and the embryos were immediately dissected. Embryo heads were fixed with 4% paraformaldehyde in phosphate-buffered saline (PBS) for 16 hours at 4 °C, then incubated in gradient sucrose solutions in PBS for 12–24 hours and embedded in OCT compound (Sakura Finetek). The heads were then sectioned into 10-μm slices with a cryostat (CM 1800; Leica) and permeabilized with 10 μg/ml of proteinase K (Roche Diagnostics) for 10–20 minutes at room temperature. For post-fixation and prehybridization, the specimens were hybridized with 20 nM of miRCURY LNA, including a detection probe (1 nmol), then 5‘-DIG and 3‘-DIG labeled (Exicon) at 51 °C overnight. Non-specific immuno-reactions were blocked with 10% normal sheep serum (Sigma), then the specimens were incubated with an anti-DIG alkaline phosphatase-conjugated antibody (Roche Diagnostics) overnight at 4 °C. The color reaction was developed using nitro blue tetrazolium (NBT) and 5-bromo-4chloro-3-indolyl phosphate (BCIP) as the substrates (Roche Diagnostics).

### Cell culture and transfection

mDP cells were derived from mouse dental mesenchymal cells^40,52^, and maintained in Dulbecco’s modified Eagle’s medium (DMEM)/F-12, supplemented with 10% fetal bovine serum (Gibco/Life Technologies), and 1% penicillin/streptomycin (Gibco/life Technologies) at 37 °C in a humidified atmosphere of 5% CO_2_. For western blotting and scratch assays, cells were cultured with 10 ng/ml of mouse recombinant protein PDGF-AA or PDGF-BB (#315-17-2UG, #315-18-2UG, PeproTech) for 24 hours. Constructed vectors, or mimic miRNA (miR875-5p, MSY0004937, Qiagen) or control miRNA (YM00479902, Qiagen) were transfected using a Neon Transfection System (Thermo Fisher Scientific), according to the manufacturer’s protocol.

### Construction of expression vectors

*Prrx1* and *Prrx2* expression vectors were constructed using a Gateway cloning system (Life Technologies), according to the manufacturer’s protocol. Briefly, the coding sequences of mouse *Prrx1* and *Prrx2* cDNA without stop codons were cloned into a pENTR/D-TOPO vector, then the following forward and reverse primers were used: *Prrx1*, 5′ - caccgggagaccatgacctccagcta - 3′ and 5′- cctgtacggagaggctgtcccccagga - 3′; and *Prrx2*, 5′ - caccatggacagcgcggccg - 3′ and 5′- gttcactgtgggcacctggc - 3′. Expression vectors were constructed using an LR recombination reaction (pcDNA-DEST40; Life Technologies) tagged with V5-His.

### Luciferase assay

An mir875 reporter plasmid was constructed by insertion of a -500 to +100 promoter sequence into a pGL4.15 vector (Promega). A *Pten* reporter plasmid was designed to contain the predicted miR875-5p binding region in *Pten* 3′UTR predicted by microRNA.org (http://www.microrna.org/microrna/home.do). The following forward and reverse primers were used: mir875, 5′ -aactcgagtgaagcaatattccatttgaact- 3′ and 5′ -aagatatccatacaattccagtaccttctcag- 3′; *Pten*, 5′ -aaactagcggccgctagttgttttattaaatgagagtattt- 3′ and 5′ -ctagaaatactctcatttaataaaacaactagcggccgctagttt- 3′; and *Pten-mutant*, 5′ -aaactagcggccgctagttgttttatttttactcacatatt- 3′ and 5′ -ctagaatatgtgagtaaaaataaaacaactagcggccgctagttt- 3′. An mir875 mutant reporter plasmid was constructed using a QuikChange II XL site-directed mutagenesis kit (Agilent Tech- nologies), with the following forward and reverse primers: mir875-mutant, 5′ -ttacatatctagcctagccgcattgttgtggggaaggattgatttatgttacttacccaaca- 3′ and 5′ -tgttgggtaagtaacataaatcaatccttccccacaacaatgcggctaggctagatatgtaa- 3′. These reporter vectors were transfected into mDP cells together with a mock vector, or the *Prrx1/2* expression vectors or mimic miR875-5p. A pRL-TK vector encoding *Renilla* luciferase was used as an internal control to co-transfect mDP cells. Luciferase activity was determined using a Dual-Luciferase Reporter Assay System (Promega) with a luminometer (Berthold Technologies). Activity was normalized to that of *Renilla* luciferase, which served as the internal control.

### Cell migration assay and immunohistochemistry

MDP cells were transfected with mimic miR875-5p or control miRNA, then seeded into 96-well plates with a stopper placed in each well (Cell Migration Assay Kit, Oris, CMA1-101, PLT). After 1 day of culture, the rubber stoppers were removed, then dental epithelium samples dissected from E14 mice molars or collagen beads coated with PDGF-AA were placed into areas without cells (previously occupied by the rubber stopper). To prepare the collagen beads (Koken), PDGF-AA (1 ng/µl) was added and stirred at 4 °C for 24 hours, then they were washed 5 times with PBS. After 6, 12, and 24 hours of culture, immunostaining was performed using primary antibodies against keratin 14 (SC-17104, 1:500; Santa Cruz) and vimentin (SC-7557-R1, 1:500, Santa Cruz), then incubation with species-specific secondary antibodies conjugated with Alexa 488 or Alexa 594 fluorescent dye (Life Technologies) was performed for 1 hour at room temperature. Nuclei were visualized by DAPI. Immunostaining of molars was performed using the primary antibody against PRRX1 (NBP1-06067, 1:500, Novus Biologicals). Images were captured using a C2 confocal microscope (Nikon) and analyzed with NIS-Elements AR software, version 4.00 (Nikon). The number of migrated cells was counted under a microscope.

### Scratch wound assay

MDP cells were transfected with mimic miR875-5p miRNA or negative control miRNA, then plated in 12-well plates (Falcon) at a density of 1 × 10^5^ cells/well in culture medium including either 10 ng/ml of PDGF-AA or PDGF-BB. Next, the cells were scratched with a 200-µl filter tip and the resulting debris was removed by gentle washing with medium, then the scratched cells were placed in an incubator. Images of closed wounds were acquired by microscopy and analyzed using ImageJ software (National Institute of Health, Bethesda, MD, USA).

### Cell proliferation assay

MDP cells were transfected with mimic miR875-5p and seeded into 96-well plates. Proliferation was measured after culturing for 1, 3, and 5 days using a Cell Counting Kit (CCK)-8 (Dojindo Laboratories), according to the manufacturer’s protocol.

### Western blotting

Total protein from cells was extracted using CelLytic M (Sigma-Aldrich) supplemented with a 1% protease inhibitor cocktail (Sigma-Aldrich) and 1 mM phenylmethylsulfonyl fluoride (Sigma-Aldrich). The protein concentration was determined using a Pierce BCA Protein Assay kit (Thermo Fisher Scientific). Next, 20 μg of protein was separated from each sample by loading in a 4-12% SDS-polyacrylamide gel (NuPAGE, Invitrogen), then transferred to a PVDF membrane (Life Technologies), and incubated with PTEN (#5384 S, 1:500; Cell Signaling Technology) and GAPDH (#5174 S, 1:500; Cell Signaling Technology) antibodies, followed by incubation with horseradish peroxidase-conjugated secondary antibodies. The membrane was developed using ECL Plus reagent (Thermo Fisher Scientific). For detection of Akt phosphorylation, cells were washed twice with 1 mM of ice-cold sodium orthovanadate (Sigma-Aldrich) in PBS before lysing with CelLytic M, then Akt (#4691 S, 1:500, Cell Signaling Technology) and P-Akt (#2965 S, 1:500, Cell Signaling Technology) were used for signal detection.

### Statistical analysis

All experiments were repeated at least 5 times to confirm reproducibility. Statistical significance was determined using a two-tailed unpaired Student’s t test with Prism 6 (GraphPad Software). One-way ANOVA was utilized for quantification between multiple groups. Differences with P values <0.05 were considered to be statistically significant.

## Supplementary information

Supplementary information
