# Supplementary material for: microRNA-875-5p plays critical role for mesenchymal condensation in epithelial-mesenchymal interaction during tooth development
Source: Sci Rep. 2020 Mar 18;10:4918. doi: 10.1038/s41598-020-61693-w (PMC7080778; doi:10.1038/s41598-020-61693-w)

Supplemental information

microRNA-875-5p plays critical role for mesenchymal condensation in epithelial-mesenchymal interaction during tooth development

Funada Keita, Yoshizaki Keigo, Miyazaki Kanako, Han Xue, Yuta Tomomi, Tian Tian, Mizuta Kanji, Fu Yao, Iwamoto Tsutomu, Yamada Aya, Takahashi Ichiro and Fukumoto Satoshi

Supplemental information

Supplemental Table 1. Tooth specific TSS identified in CAGE analysis

Chrm	chrm start	chrm end	strand	symbol
chr01	138156923	138157039	-	Gpr25
chr02	35960928	35961080	-	Lhx6
chr02	80287218	80287582	-	Frzb
chr03	128916842	128916884	+	Pitx2
chr03	153994942	153995071	+	*
chr03	153993783	153993892	+	*
chr03	86355146	86355245	-	*
chr03	153995256	153995345	+	*
chr05	38215778	38215829	-	Msx1
chr05	104728305	104728308	+	Ibsp
chr05	91015724	91015742	+	*
chr05	88885010	88885024	+	Ambn
chr06	4644082	4644189	-	*
chr06	51116326	51116477	-	*
chr06	66214115	66214192	+	*
chr06	8899796	8899861	+	Nxph1
chr06	77193835	77193930	+	Lrrtm1
chr07	51562258	51562320	-	Clec11a
chr10	27336836	27336849	-	Lama2
chr11	56825176	56825312	+	Gria1
chr12	80749564	80749634	+	*
chr12	57794217	57794300	-	*
chr12	74645939	74646016	+	*
chr12	74614501	74614674	-	*
chr13	43711323	43711410	+	Rnf182
chr14	124059316	124059425	+	Itgbl1
chr15	35591507	35591567	-	miR599
chr15	35591507	35591567	-	miR875
chr16	57549095	57549175	+	Filip1l
chr17	44951435	44951611	-	Runx2

Supplemental information

Supplemental Table 2. Potential binding transcriptional factors to the promoter region of mir875

Model ID	Model name	Score	Relative score	Start	End	Strand	predicted site sequence
MA0032.1	FOXC1	7.472	1.000022	415	422	-1	GGTAAGTA
MA0081.1	SPIB	10.470	1.000015	205	211	1	AGAGGAA
MA0152.1	NFATC2	11.360	1.000012	607	613	1	TTTTCCA
MA0156.1	FEV	12.047	1.000010	186	193	-1	CAGGAAAT
MA0151.1	ARID3A	9.844	0.999991	332	337	1	ATTAAA
MA0151.1	ARID3A	9.844	0.999991	349	354	-1	ATTAAA
MA0151.1	ARID3A	9.844	0.999991	437	442	1	ATTAAA
MA0075.1	Prrx2	9.124	0.999985	251	255	-1	AATTA
MA0075.1	Prrx2	9.124	0.999985	394	398	-1	AATTA
MA0075.1	Prrx2	9.124	0.999985	395	399	1	AATTA
MA0075.1	Prrx2	9.124	0.999985	457	461	-1	AATTA
MA0075.1	Prrx2	9.124	0.999985	465	469	-1	AATTA
MA0598.1	EHF	13.801	0.999533	206	213	-1	CCTTCCTC
MA0442.1	SOX10	8.625	0.987357	386	391	1	CATTGT
MA0063.1	Nkx2-5	8.996	0.987069	456	462	1	ATAATTG
MA0033.1	FOXL1	8.361	0.983960	154	161	-1	TAGACATA
MA0133.1	BRCA1	7.954	0.980976	422	428	1	CCAACAC
MA0087.1	Sox5	10.075	0.969330	387	393	1	ATTGTTG
MA0151.1	ARID3A	9.015	0.967649	232	237	-1	ATCAAA

Supplemental information

Supplemental Table 3. Up and down regulated genes in mDP cells transfected with mir875-5p

Up-regulated genes

Gene	Fold Change
5830417I10Rik	207.00
1700047I17Rik2	147.10
Hist1h4k	111.19
Gm16209	45.70
Tmem181b-ps	28.42
Gm6793	21.80
Gm14200	20.69
Pcnp	16.29
Cdk2ap1	9.32
Gm14934	9.29
Zmym5	8.23
Snord22	6.36
SNORD31	6.36
Vcp-rs	5.72
Gm9703	5.08
Gm11628	4.99
Gm8163	4.33
SNORD30	4.10
AC123853.2	4.09
Hp	4.08
Acss2	3.92
3110057O12Rik	3.88
1100001G20Rik	3.74
Scd2	3.65

Down-regulated genes

Gene	Fold Change	Gene	Fold Change
Cxcl10	0.01	Gm14017	0.11
Gbp3	0.01	Parp12	0.13
Isg15	0.01	Ddx58	0.13
Irf7	0.01	Trim12c	0.13
Ifi27l2a	0.02	Herc6	0.14
Gm6109	0.02	BC006779	0.14
Bst2	0.03	Ube2l6	0.14
Samd9l	0.05	Adar	0.14
Stat1	0.05	Psmb9	0.14
D5Ertd579e	0.06	Parp9	0.15
Xaf1	0.06	H2-K2	0.15
Mndal	0.06	Stat2	0.16
Ifi203	0.06	Tap1	0.16
Parp14	0.07	Dtx3l	0.17
Gm11847	0.07	H2-D1	0.17
Gm5687	0.08	Ifitm3	0.17
Capza1	0.08	Trim21	0.18
Irf9	0.09	Ahcy	0.18
Gbp2	0.09	B2m	0.18
H2-Q4	0.10	Cox6a2	0.18
Tor3a	0.10	Lgals3bp	0.19
Irgm1	0.10	Ifi35	0.19
AW011738	0.10	Psmb8	0.20

Supplemental information

Supplemental Figure 1. The full-length blots of western blotting

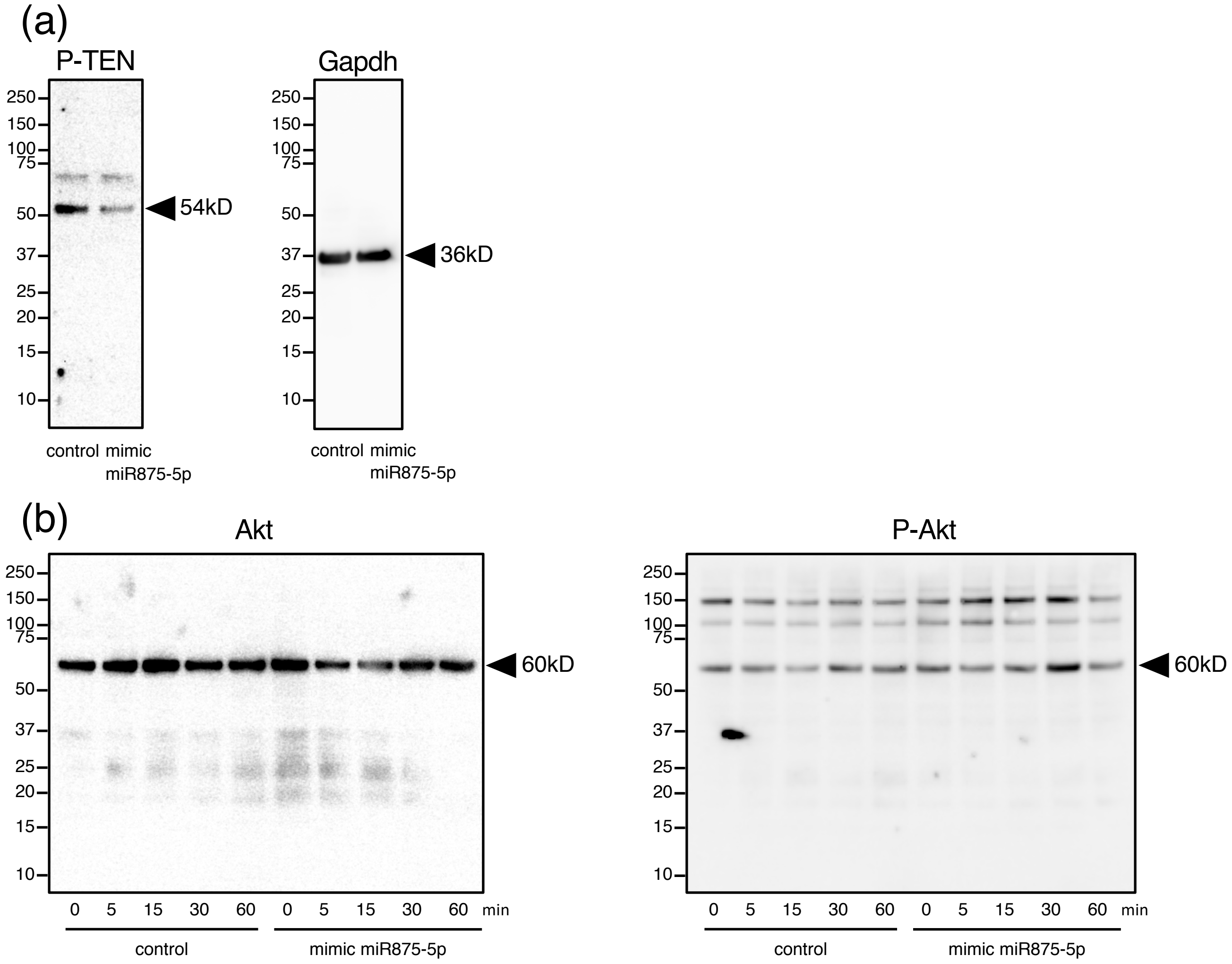

Supplemental information

Supplemental Figure 2. Pten and stat1 expression in tooth development

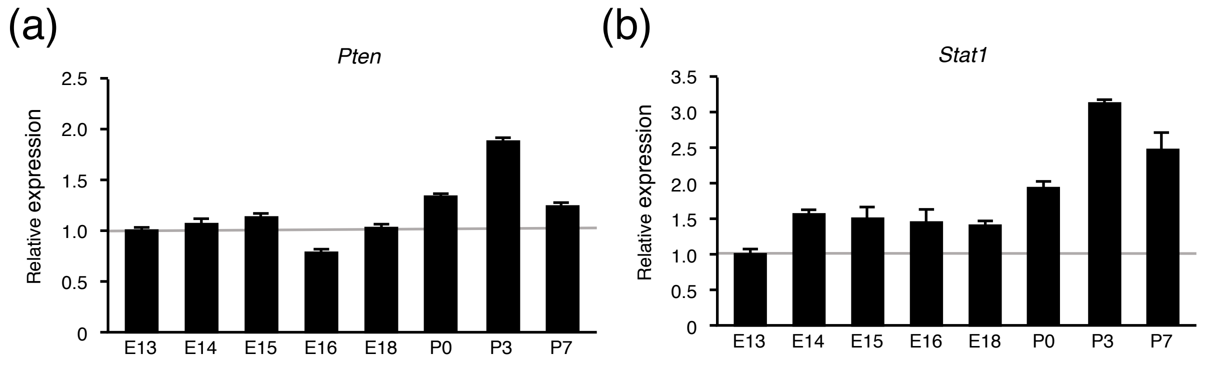

Supplement: Supplementary file 1 — Supplementary information [file 41598_2020_61693_MOESM1_ESM.pdf]
